# Sex-specific associations of serum short-chain fatty acids with glycaemic control: an Italian cross-sectional study in adults with type 1 diabetes

**DOI:** 10.1136/bmjopen-2024-096994

**Published:** 2025-03-24

**Authors:** Jumana Abuqwider, Dominic Salamone, Giuseppe Scidà, Alessandra Corrado, Giuseppina Costabile, Delia Luongo, Giovanni Annuzzi, Angela Rivellese, Lutgarda Bozzetto

**Affiliations:** 1 Clinical Medicine and Surgery, University of Naples Federico II, Naples, Italy; 2 National Research Council, Institute of Biostructures and Bioimaging, Napoli, Italy

**Keywords:** DIABETES & ENDOCRINOLOGY, NUTRITION & DIETETICS, Cross-Sectional Studies

## Abstract

**Objective:**

Short-chain fatty acids (SCFA) play a role in modulating glucose metabolism and are influenced by diet. Alterations in the SCFA-producing microbial ecosystem in individuals with type 1 diabetes (T1D) may contribute to impaired glycaemic control. This study investigated the relationships between serum SCFA levels, blood glucose control, and dietary habits in adults with T1D.

**Design:**

Observational study.

**Setting:**

The study was conducted at the diabetes outpatient clinic of Federico II University Teaching Hospital, Naples, Italy.

**Population:**

The study included 198 adults with T1D (100 men and 98 women), aged 18–79 years.

**Main outcome measures:**

Serum SCFA levels, blood glucose control, assessed by glycated haemoglobin (HbA1c) and continuous glucose monitoring (CGM) metrics, and dietary intake from a 7-day food record.

**Results:**

SCFA levels showed significant sex-specific differences (p<0.05). Therefore, to evaluate relationships between SCFA levels, glycaemic control and dietary habits, SCFA levels were categorised into sex-specific tertiles, and results were adjusted for age and body mass index. HbA1c and CGM metrics did not vary significantly across tertiles of acetate and butyrate. However, in women, higher propionate levels were associated with better glycaemic control, reflected by a greater percentage of glucose time-in-range (70–180 mg/dL) (66.2±12.3% vs 56.9±16.7%, low tertile; p=0.014), lower time-above-range (>180 mg/dL) (32.2±12.6% vs 41.2±17.2%, low tertile; p=0.011) and improved glucose management indicator (7.1±0.6% vs 7.5±0.6%, low tertile; p=0.027). Regarding eating habits, higher acetate tertiles were associated with higher intakes of total fat (p=0.041), polyunsaturated fatty acids (p=0.049) and monounsaturated fatty acids (p=0.021) in men only.

**Conclusion:**

These findings reveal a sex-specific association between serum propionate levels and blood glucose control in women with T1D. Importantly, this relationship appears independent of dietary factors.

**Trial registration number:**

NCT05936242.

**STRENGTHS AND LIMITATIONS OF THIS STUDY**

- The study provides a comprehensive evaluation of glycaemic control by integrating glycated haemoglobin measurements with continuous glucose monitoring metrics, offering a more detailed and dynamic assessment.
- Statistical analyses are rigorously adjusted for key confounders, such as age and body mass index, enhancing the reliability of the observed associations between short-chain fatty acids (SCFA), glycaemic control and dietary habits.
- The cross-sectional design of the study restricts the ability to infer causality between SCFA, glycaemic control and dietary factors, leaving the direction of these relationships uncertain.

## Introduction

 The gut microbiota plays a pivotal role in regulating glucose metabolism and may contribute to the development of insulin resistance and type 2 diabetes (T2D).^1 2^ Observational and interventional studies in humans have highlighted the potential role of gut microbiota and short-chain fatty acid (SCFA) in glucose metabolism regulation, particularly in individuals with high cardiometabolic risk and T2D.^1^ However, the relationship between gut microbiota and type 1 diabetes (T1D) is less clearly understood.^3^ Evidence suggests that individuals with T1D exhibit a more pronounced dysbiosis, characterised by a reduced abundance of SCFA-producing bacteria compared with healthy controls.^4 5^ This altered microbial composition may contribute to impaired glycaemic control in people with T1D.^3^

SCFA—primarily acetate, propionate and butyrate—are critical modulators of metabolic pathways and exert both gastrointestinal and systemic effects on glucose regulation.^6^ In vivo and in vitro studies have shown that SCFA stimulate the secretion of glucagon-like peptide-1 (GLP-1) and peptide YY (PYY) by L cells, hormones that enhance satiety, reduce appetite and lower food intake, thereby contributing to body weight regulation.^7^ SCFA also activate G-protein-coupled receptors (GPR41 and GPR43) in the liver, skeletal muscle and adipose tissue, improving both hepatic and peripheral insulin sensitivity.^7^

Dietary habits, especially fibre intake, are key determinants of plasma and faecal SCFA levels.^8 9^ Human studies indicate that elevated serum and faecal SCFA levels, particularly propionate and butyrate, are associated with higher fibre consumption and improved glucose metabolism in both healthy individuals and those with T2D.^9^ Interventional studies in populations at high cardiometabolic risk have shown that diets rich in whole grains (cereal-derived fibre) elevate plasma propionate levels and enhance postprandial insulin responses. Similarly, a Mediterranean diet, characterised by diverse fibre sources, has been shown to elevate postprandial butyrate concentrations and insulin sensitivity.^10 11^

Despite these insights, data on the relationship between SCFA levels, dietary habits and glucose metabolism in T1D remain limited. Existing studies predominantly focus on faecal SCFA levels.^12^,^14^ Serum SCFA levels may provide a more direct indication of their effects at potential sites of action, offering critical insights into their relationship with metabolic health.^15^

This study aimed to evaluate the associations between serum SCFA levels, blood glucose control and dietary habits in adults with T1D.

## Materials and methods

### Study design and recruitment

This cross-sectional observational study recruited individuals with T1D from the diabetes outpatient clinic at Federico II University Teaching Hospital (Naples, Italy) between July 2021 and November 2022. Participants were aged 19–79 years, were of both sexes, were users of continuous glucose monitoring (CGM) devices and were on either insulin multiple daily injections or continuous subcutaneous insulin infusion. Inclusion criteria required a diagnosis of T1D for at least 1 year and the absence of chronic or acute comorbidities, coeliac disease, pregnancy, or antibiotic, prebiotic or probiotic use within the 3 months preceding recruitment.

Eligible patients were invited to participate in the study via phone call 1 week before their scheduled annual visit for the screening of chronic complications. Participants were instructed to complete a 7-day food diary using a standardised form. Completed food diaries were returned by 137 participants, reviewed by a trained dietitian to identify and correct any errors or omissions and analysed using the Metadieta software (V.4.5, Meteda s.r.l., Italy). All foods were entered into the software using the Italian Food Composition Databases,^16^ and the 7-day average nutrient intake for each participant was calculated.

All participants gave their written informed consent. The study protocol was registered under clinical trial number NCT05936242.

### Anthropometric measurements and blood glucose control

During the hospital visit, body weight was measured using a mechanical balance with participants wearing light clothing and no footwear, and height was recorded using a stadiometer. Body mass index (BMI) was calculated as weight (kg) divided by the square of height (m²). Blood samples were collected in the morning after an overnight fast, and all biochemical analyses were conducted at a central laboratory.

Glycaemic control was evaluated using glycated haemoglobin (HbA1c) (high-performance liquid chromatography, standardised to International Federation of Clinical Chemistry and Laboratory Medicine (IFCC) and CGM metrics. CGM data relative to the 2 weeks preceding the visit were downloaded from the dedicated platforms of the systems used by the patients (FreeStyle Libre2, n=57; Eversense, n=3; Dexcom G6, n=51; Guardian3, n=35; Guardian4, n=52) and the following CGM metrics were analysed: percentage of time spent in the interstitial glucose range between 70 and 180 mg/dL (time-in-range, TIR), above 180 mg/dL (time-above-range, TAR), below 70 mg/dL (time-below-range, TBR) and the glucose management indicator (GMI). According to an international consensus, optimal glycaemic control is defined as a TIR>70%, TAR<25% and TBR<5%.^17^

### SCFA analysis

SCFA acetic, propionic, and butyric acid were extracted from serum and quantified using gas chromatography coupled with a flame ionisation detector (GC/FID) (Dani, Analitica Instruments S.p.A., Milan, Italy). The analysis employed a megabore column compatible with aqueous solvents, following the method described by Remesy and Demigne.^18^

Serum samples were deproteinised by the addition of metaphosphoric acid. In these conditions, positively charged proteins acted as polycations, co-precipitating with metaphosphoric acid. Proteins were then removed by centrifugation, and SCFA analysis was carried out on the supernatant by GC/FID. Subsequently, isovaleric acid was added as an internal standard. A stock solution of acetic, propionic and butyric acid at varying concentrations (low, medium and high) was employed to establish the calibration curve. A sample with known concentrations of the individual SCFA was injected at both the beginning and the end of each experimental day as a quality control to evaluate both concentrations and retention times. The coefficient of variation intra-assay was 2.2, 2.0 and 1.2%, and interassay 2.9, 2.0 and 1.8% for acetate, propionate and butyrate, respectively.

### Statistical analysis

A sample size of 134 participants was estimated to detect a 20% difference in glycaemic control based on serum acetate levels (power=90%, α=0.05).^19^ Data are presented as means±SD unless otherwise specified. The Kolmogorov-Smirnov test was used to assess the normality of the distribution for each variable. Variables that did not exhibit a normal distribution were log-transformed prior to analysis. Differences between sexes were evaluated using unpaired t-tests for continuous variables and χ^2^ tests for categorical variables. To assess the associations between SCFA, glycaemic control and dietary habits, participants were stratified into sex-specific tertiles of acetic, propionic and butyric acid concentrations. Differences across tertiles were analysed using one-way analysis of variance, with linear trend analysis conducted to identify patterns across the tertiles. All variables were adjusted for potential confounders, specifically age and BMI, using a general linear model for univariate analysis. Statistical analyses were conducted using SPSS software V.29 (SPSS/PC). Sample size was calculated using the ‘pwr’ package, and boxplots were visualised using the ggplot2 package in R (V.4.3.2).

### Patient and public involvement

Patients or the public were not involved in the design, or conduct, or reporting, or dissemination plans of our research.

## Results

### Population characteristics

Table 1 summarises the anthropometric and clinical characteristics of the study population comprising 198 participants (100 men and 98 women). The mean age was 38.8±13.7 years, with a BMI of 25.7±4.3 kg/m². HbA1c levels were 7.5±0.9%, while the TIR was 63.6±15.9% and the GMI 7.2±0.6%. Sex-specific stratification revealed that women had significantly higher HbA1c, acetic acid and total SCFA levels compared with men (p<0.05). Additionally, men exhibted a higher use of antihypertensive drugs (table 1).

**Table 1:** Main characteristics, blood glucose control and serum SCFA levels in the whole population and according to sex

	Whole population (n=198)	Men(n=100)	Women(n=98)	P value*
Age (years)	38.8±13.7	39.9±14.4	37.7±13.1	0.271
BMI (kg/m^2^)	25.7±4.3	26.0±3.8	25.4±4.8	0.329
HbA1c (%)	7.5±0.9	7.2±0.9	7.7±0.9	**0.003**
HbA1c (mmol/mol)	58.0±10.0	55.6±9.9	60.4±9.7	**0.002**
GMI (%)	7.2±0.6	7.1±0.6	7.3±0.6	0.108
TIR_70–180mg/dl_ (%)	63.6±15.9	65.5±15.7	61.8±15.9	0.156
TAR_>180mg/dl_ (%)	34.0±16.4	31.9±16.1	36.1±16.6	0.126
TBR_<70mg/dl_ (%)	2.4±3.2	2.6±3.4	2.3±3.1	0.574
Insulin therapy				
Closed loop	95	49	46	0.640
Open loop	67	31	36	
Multiple daily injections	36	20	16	
Lipid-lowering drugs (%)	25.3	27.0	23.5	0.623
Antihypertensive drugs (%)	15.2	20.0	10.2	**0.049**
Acetic acid (µmol/L)	311.5±70.2	300.8±66.0	322.5±72.9	**0.029**
Propionic acid (µmol/L)	27.9±8.1	27.0±7.9	28.9±8.3	0.096
Butyric acid (µmol/L)	21.6±7.5	21.1±7.1	22.1±7.8	0.374
Total SCFA (µmol/L)	361.2±73.3	349.0±69.9	373.6±74.9	**0.017**

Data are expressed as mean±SD.

* Men versus women.

BMIbody mass indexGMIglucose management indicatorHbA1cglycated haemoglobinHDLhigh density lipoproteinLDLlow density lipoproteinSCFAshort-chain fatty acidsTARtime above rangeTBRtime below rangeTIRtime in range

### SCFA and blood glucose control

Building on the observed differences between men and women, we conducted a more detailed analysis of SCFA levels by stratifying participants into sex-specific tertiles and adjusting for age and BMI, both known to influence SCFA concentrations.

**Figure 1:**
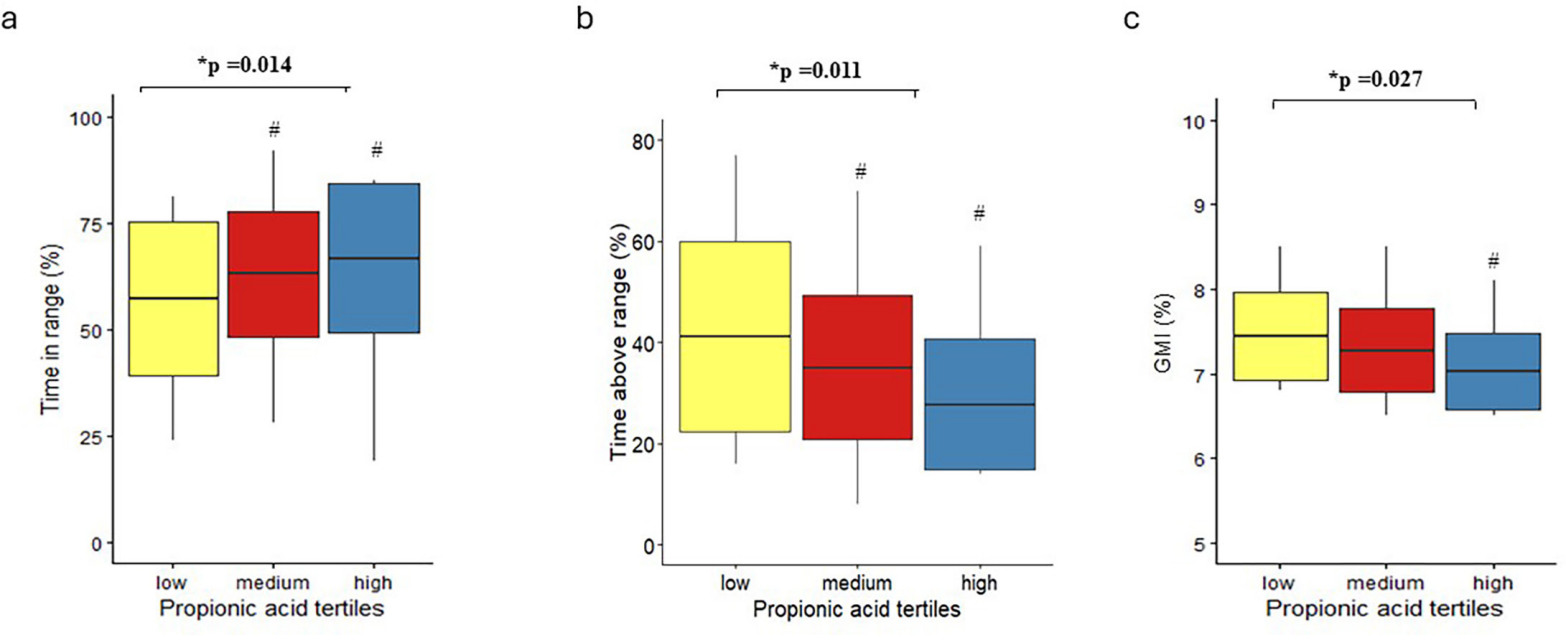
Glucose time-in-range: 70–180 mg/dL (a), time-above-range: >180 mg/dL (b) and glucose management indicator (GMI) (c) according to tertiles of serum propionic acid levels in the female study participants. Boxplots display the mean (central line), one SD (lower and upper bounds of the box) and full range (vertical line). *General linear model for univariate analysis adjusted for age and body mass index. #p<0.05 vs low tertile, Bonferroni post-hoc analysis.

No significant differences in HbA1c, GMI, TIR, TAR or TBR were observed across tertiles of acetate or butyrate levels (onlinesupplemental tables 1  2). Conversely, in women, compared with the low tertile, adjusting for age and BMI, the high tertile of propionate was significantly associated with a 16% higher TIR_70–180mg/dl_ (p=0.014), a 22% lower TAR_>180mg/dl_ (p=0.011) and a 5% lower GMI (p=0.027) (table 2, figure 1). Notably, no significant differences were observed in TBR across tertiles (table 2). No differences were detected in the distribution of insulin administration methods (closed-loop systems, open-loop systems or multiple daily injections) or the use of lipid-lowering and antihypertensive medications (table 2).

**Table 2:** Anthropometric parameters, therapy and blood glucose control according to propionic acid tertiles stratifying the cohort by sex

Men	Low tertile(<23.2 µmol/L)(n=33)	Medium tertile(23.2–30.8 µmol/L)(n=34)	High tertile(>30.8 µmol/L)(n=33)	P value for trend	P valueANOVA	P value adjusted for age and BMI
Age (years)	41.8±15.2	39.1±13.6	38.4±14.7	0.345	0.685	
BMI (kg/m^2^)	25.9±4.2	25.8±3.8	26.4±3.5	0.634	0.830	
HbA1c (%)	7.4±1.2	7.1±0.5	7.3±1.0	0.696	0.650	0.553
HbA1c (mmol/mol)	56.8±12.7	54.5±6.1	55.9±10.5	0.741	0.667	0.570
GMI (%)	7.2±0.7	7.1±0.6	7.3±0.5	0.642	0.632	0.413
TIR_70–180 mg/dl_ (%)	63.5±17.8	68.1±16.6	64.7±12.9	0.807	0.576	0.436
TAR_>180 mg/dl_ (%)	33.2±18.7	29.6±16.5	33.2±13.4	0.666	0.999	0.526
TBR_<70 mg/dl_ (%)	3.3±5.2	2.3±2.0	2.1±2.1	0.243	0.464	0.200
Insulin therapy (M/OL/CL)	5/15/13	9/6/19	6/9/18		0.165	
Lipid-lowering drugs (%)	24.2	33.3	24.2		0.792	
Antihypertensive drugs (%)	24.2	17.6	18.1		0.699	
**Women**	**Low tertile**(**<25.1 µmol/L) (n=32**)	**Medium tertile(25.1–32.1 µmol/L**)(**n=33**)	**High tertile**(**>32.1 µmol/L**)(**n=33**)	**P value for trend**	**P value ANOVA**	**P value adjusted for age and BMI**
Age (years)	39.2±12.3	36.1±14.4	37.3±12.6	0.575	0.645	
BMI (kg/m^2^)	24.5±4.6	26.7±5.5	24.7±3.9	0.876	0.149	
HbA1c (%)	7.6±1.0	7.7±0.9	7.7±0.8	0.573	0.732	0.816
HbA1c (mmol/mol)	59.4±10.7	60.9±10.1	60.5±8.3	0.682	0.846	0.919
GMI (%)	7.5±0.6	7.2±0.5	7.1±0.6^*^	**0.044**	0.111	**0.027**
TIR_70–180 mg/dl_ (%)	56.9±16.7	64.3±15.5	66.2±12.3^*^	**0.025**	0.062	**0.014**
TAR_>180 mg/dl_ (%)	41.2±17.2	32.8±16.4^*^	32.2±12.6^*^	**0.042**	0.077	**0.011**
TBR_<70 mg/dl_ (%)	1.9±2.8	2.9±4.2	2.1±1.7	0.892	0.461	0.475
Insulin therapy (M/OL/CL)	4/14/15	5/13/15	7/9/16		0.704	
Lipid-lowering drugs (%)	25.0	21.2	21.9		0.927	
Antihypertensive drugs (%)	6.3	12.1	12.5		0.653	

Data are expressed as mean±SD.

* p<0.05 versus low tertile, Bonferroni post-hoc analysis.

ANOVAanalysis of varianceBMIbody mass indexCLclosed loopGMIglucose management indicatorHbA1cglycated haemoglobinMmultiple daily injectionsOLopen loopTARtime above rangeTBRtime below rangeTIRtime in range

### SCFA and dietary habits

**Figure 2:**
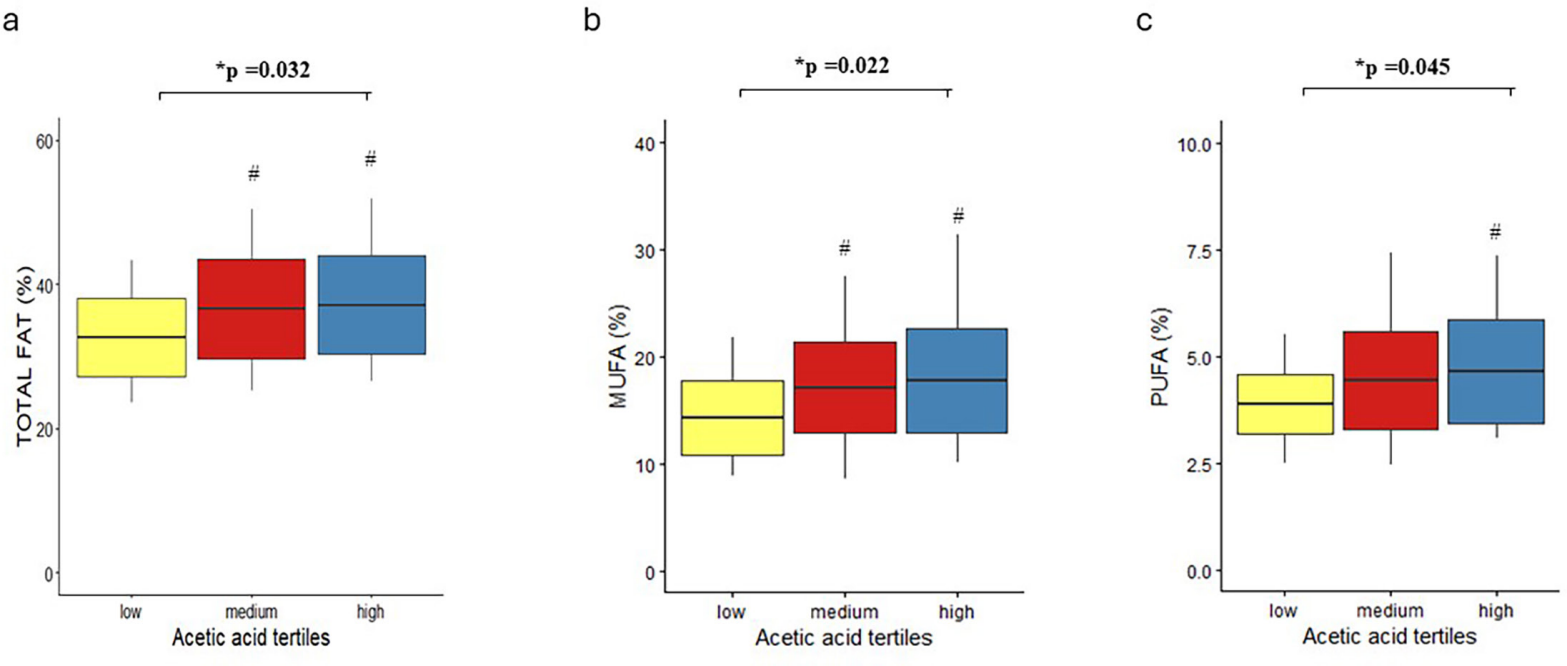
Dietary intakes of total fat (a), monounsaturated fat (MUFA) (b) and polyunsaturated fat (PUFA) (c) according to tertiles of serum acetic acid levels in the male study participants. Boxplots display the mean (central line), one SD (lower and upper bounds of the box) and full range (vertical line). *General linear model for univariate analysis adjusted for age and body mass index. #p<0.05 versus low tertile, Bonferroni post-hoc analysis.

Analysis of the 7-day food records showed that women consumed less total energy, a higher proportion of energy from polyunsaturated fatty acids (PUFA) and more dietary fibre than men (table 3). In men, higher acetate levels positively related to energy derived from total fat, PUFA and monounsaturated fatty acids (MUFA) (table 4, figure 2). In women, acetic acid levels negatively correlated with the percentage of energy derived from total proteins and animal proteins, with significant differences between medium and higher tertiles (table 4). In women, the medium tertile of propionic acid was associated with lower energy consumption from total fat and higher from carbohydrates (online supplemental table 3). Finally, in men, the medium tertile of butyric acid was associated with higher consumption of energy from total fat (online supplemental table 4).

**Table 3:** Daily dietary composition (7-day food record) in the whole population and according to sex

	Whole population (n=137)	Men (n=69)	Women (n=68)	P value*
Energy (kcal)	1438±380	1585±397	1289±296	**<0.001**
Protein (%)	19.7±3.3	20.1±3.5	19.4±3.1	0.213
- Vegetables (%)	6.9±1.2	6.8±1.5	6.9±1.9	0.789
- Animals (%)	11.3±3.6	11.7±3.6	11.0±3.7	0.213
Total Fat (%)	36.4±6.1	35.5±6.7	37.3±5.4	0.086
- SFA (%)	11.2±3.1	11.1±3.5	11.2±2.6	0.890
- MUFA (%)	16.9±4.0	16.5±4.4	17.3±3.6	0.189
- PUFA (%)	4.5±1.1	4.3±1.1	4.7±1.2	**0.037**
Carbohydrates (%)	44.1±6.2	44.7±6.4	43.6±6.1	0.306
Simple sugars (%)	12.4±4.8	12.1±5.1	12.7±4.4	0.440
Fibre (g/1000 kcal)	11.4±4.0	10.6±3.4	12.3±4.4	**0.014**

Data are expressed as mean±SD.

* Men versus women.

MUFAmonounsaturated fatty acidsPUFApolyunsaturated fatty acidsSFAsaturated fatty acids

**Table 4:** Daily dietary composition (7-day food diary) according to acetic acid tertiles stratifying the cohort by sex

MEN	Low tertile(<261.9 µmol/L) (n=21)	Medium tertile(261.9–324.5 µmol/L) (n=26)	High tertile(>324.5 µmol/L) (n=22)	P value for trend	P value ANOVA	P value adjusted for age and BMI
Energy (kcal)	1595±376	1599±477	1567±324	0.820	0.969	0.893
Protein (%)	20.7±3.8	19.3±3.2	20.3±3.7	0.711	0.360	0.356
- Vegetables (%)	7.2±1.2	6.6±1.3	6.8±1.8	0.350	0.363	0.291
- Animals (%)	11.7±3.5	11.5±3.2	12.0±4.3	0.787	0.911	0.901
Total Fat (%)	32.5±5.3	36.6±6.9^*^	37.1±6.9^*^	**0.023**	**0.041**	**0.032**
- SFA (%)	10.6±4.6	11.5±2.8	11.1±3.5	0.506	0.615	0.516
- MUFA (%)	14.3±3.5	17.1±4.2^*^	17.8±4.9^*^	**0.009**	**0.021**	**0.022**
- PUFA (%)	3.9±0.7	4.4±1.2	4.7±1.2^*^	**0.018**	**0.049**	**0.045**
Carbohydrates (%)	47.0±6.1	44.3±5.7	42.8±7.1	**0.031**	0.090	0.093
Simple sugars (%)	13.8±6.2	11.9±4.6	10.9±4.1	0.052	0.145	0.224
Fibre (g/1000 kcal)	11.1±3.6	9.9±2.3	11.0±4.1	0.955	0.376	0.105
**WOMEN**	**Low tertile**(**<291.3 µmol/L) (n=26**)	**Medium tertile(291.3–358.4 µmol/L) (n=21**)	**High tertile**(**>358.4 µmol/L) (n=21**)	**p for trend**	**p-valueANOVA**	**p-value adjusted for age and BMI**
Energy (kcal)	1360±299	1259±323	1207±239	0.077	0.196	0.237
Protein (%)	19.1±2.5	21.1±3.5	17.9±2.5^†^	0.232	**0.002**	**<0.001**
- Vegetables (%)	7.3±1.7	6.5±1.9	6.9±2.0	0.390	0.356	0.229
- Animals (%)	10.2±3.0	13.3±4.1	9.6±3.1^†^	0.762	**0.001**	**<0.001**
Total Fat (%)	37.3±4.0	36.9±6.1	37.5±6.3	0.938	0.955	0.959
- SFA (%)	11.5±2.6	11.4±2.7	10.5±2.7	0.261	0.444	0.420
- MUFA (%)	17.2±2.7	17.3±3.7	17.8±4.4	0.644	0.980	0.834
- PUFA (%)	4.8±1.1	4.7±1.2	4.7±1.1	0.844	0.980	0.960
Carbohydrates (%)	43.8±4.9	42.1±6.9	44.9±6.6	0.610	0.338	0.247
Simple sugars (%)	13.8±3.6	12.0±3.8	11.8±5.6	0.121	0.241	0.402
Fibre (g/1000 kcal)	13.2±4.0	12.3±4.7	11.3±4.6	0.149	0.350	0.319

Data are expressed as mean±SD.

* p<0.05 versus low tertile.

† p<0.05 versus medium tertile, Bonferroni post-hoc analysis.

ANOVAanalysis of varianceMUFAmonounsaturated fatty acidsPUFApolyunsaturated fatty acidsSFAsaturated fatty acids

## Discussion

This study investigated the potential associations between serum SCFA, blood glucose control and dietary habits in individuals with T1D, addressing a gap in the current literature. We focused on sex-specific classifications while adjusting for age and BMI, as SCFA levels are known to differ between men and women, with both age and BMI influencing SCFA concentrations.

Regarding blood glucose control, the key findings of our study are as follows: (1) no significant differences in HbA1c, TIR, TAR and TBR were observed across tertiles of butyrate and acetate levels in both men and women. This suggests that, within our study population, these specific SCFA may not exert a significant effect on blood glucose control. (2) In contrast, a notable sex-specific effect was observed concerning propionate levels. Women in the highest tertiles of propionate exhibited significantly higher percentages of TIR, lower percentages of TAR, and lower GMI levels, even after adjusting for potential confounders such as age and BMI. The observed differences in TIR were not only statistically significant but also clinically meaningful, with a difference of approximately 9 percentage points of TIR, along with a significant reduction in GMI values. On the other hand, the lack of differences in HbA1c values could be due to the fact that they reflected data from the preceding 2–3 months, while the 2-week CGM data could capture shorter-term fluctuations in glucose control missed by HbA1c measurements.

As noted in the introduction, no prior studies have specifically investigated the relationship between serum SCFA levels and blood glucose control in T1D. Furthermore, there is no direct evidence in the existing literature to suggest that propionate influences glucose metabolism differently in men and women. However, findings from studies in humans, animal models and in vitro experiments indicate that propionate may improve glucose metabolism through mechanisms such as enhanced glycolysis, reduced hepatic glucose production, improved insulin sensitivity and reduced inflammation.^20^ These mechanisms may also apply to T1D and could explain our findings, where propionate showed a stronger association with glycaemic control in women. The more pronounced effect observed in women might be related to their poorer baseline glycaemic control, as reported in table 1. Sex-specific differences in the effects of propionate may also be driven by hormonal factors, particularly oestrogen, which may influence gut microbiota composition and function. These hormonal effects could lead to distinct metabolic outcomes, including variations in SCFA production, such as propionate.^21^ It is also plausible that propionate exerts a more pronounced impact on glucose metabolism in women, a hypothesis that requires further exploration in future studies. It is important to acknowledge that the cross-sectional design of this study limits the ability to establish causality. Although unlikely, the possibility that improved glycaemic control might result in increased propionate levels cannot be entirely excluded.

In contrast to propionate, neither acetate nor butyrate demonstrated significant associations with glycaemic control metrics in our study. This aligns with prior research, which has shown inconsistent effects of these SCFA on metabolic outcomes. For instance, Canfora *et al* found that acetate promotes lipid oxidation and energy expenditure but has a less pronounced effect on glycaemic control compared with propionate.^7^ Similarly, while butyrate has been shown to enhance gut barrier function and reduce inflammation in animal models,^22^ its direct influence on glycaemic control remains unclear.^23^

Given the recognised role of dietary habits in modulating SCFA levels, we explored this relationship in our cohort. Analysis of 7-day food records revealed significant sex-specific dietary patterns. Women consumed less total energy, derived a higher proportion of energy from PUFA and consumed more dietary fibre compared with men. These findings are consistent with previous studies suggesting that women typically have higher fibre intake and lower caloric consumption than men.^24^

Regarding the relationship between SCFA levels and dietary variables, our results were again sex-specific and somewhat heterogeneous. A notable finding was the positive correlation between fat consumption, particularly PUFA and MUFA, and acetic acid levels in men. This suggests that a higher intake of healthy fats, such as PUFA and MUFA, may promote acetic acid production. Previous research by Costantini *et al* supports this notion, showing that PUFA can positively influence gut microbiota composition by promoting beneficial bacteria such as *Bifidobacterium* and *Lactobacillus*, which enhance SCFA production, including acetic acid.^25^ However, despite these dietary associations, acetic acid levels were not linked to glycaemic control metrics in our cohort. The observed relationship between higher propionate levels and improved glycaemic control in women could not be fully explained by dietary variables alone. This suggests that additional factors, such as microbiota composition, host–microbe interactions or hormonal influences, may play a critical role. Further research is warranted to explore these factors in greater detail.

The primary strength of this study lies in its relatively large cohort of individuals with T1D and the attention given to potential sex differences, an area often neglected in similar research. However, some limitations should be noted. The cross-sectional design limits causal inference, and the lack of detailed gut microbiome data restricts our ability to fully interpret the observed relationships between SCFA levels and metabolic outcomes.

## Conclusion

In summary, these findings emphasise the importance of considering sex-specific responses in metabolic research. Propionate, in particular, shows promise as a modulator of glycaemic outcomes, especially in women with T1D. The significant improvements in TIR and reductions in TAR observed in women in the highest tertile of propionate suggest its potential as a therapeutic target for improving glycaemic control. Future studies should aim to elucidate the underlying mechanisms, investigate causal relationships and explore the potential for dietary or microbiota-based interventions to optimise SCFA production and improve metabolic outcomes in individuals with T1D.

## supplementary material

10.1136/bmjopen-2024-096994 online supplemental file 1

10.1136/bmjopen-2024-096994 online supplemental file 2

10.1136/bmjopen-2024-096994 online supplemental file 3

10.1136/bmjopen-2024-096994 online supplemental file 4

## Data Availability

Data are available upon reasonable request.
