# Supplementary material for: Sex-specific associations of serum short-chain fatty acids with glycaemic control: an Italian cross-sectional study in adults with type 1 diabetes
Source: BMJ Open. 2025 Mar 24;15(3):e096994. doi: 10.1136/bmjopen-2024-096994 (PMC11934402; doi:10.1136/bmjopen-2024-096994)
Supplement: online supplemental file 1 [file bmjopen-15-3-s001.docx]

| **Supplementary table 1**. Anthropometric parameters, therapy, and blood glucose control according to acetic acid tertiles stratifying the cohort by sex. | | | | | | | |
| --- | --- | --- | --- | --- | --- | --- | --- |
| **MEN** | | | | | | | |
|  | **Low tertile**  **(<261.9 µmol/L) (n=33)** | **Medium tertile**  **(261.9-324.5** **µmol/L) (n=34)** | **High tertile**  **(> 324.5** **µmol/L) (n=33)** | **p for trend** | **p-value**  **ANOVA** | **p-value**  **adjusted for age and BMI** |  |
| Age (Years) | 40.7±13.2 | 43.9±15.9^a^ | 34.8±12.5 | 0.093 | **0.032** |  |  |
| BMI(Kg/m^2^) | 25.9±4.1 | 26.0±3.9 | 26.1±3.4 | 0.806 | 0.970 |  |  |
| HbA1c (%) | 7.2±0.9 | 7.3±1.0 | 7.2±0.8 | 0.867 | 0.823 | 0.773 |  |
| HbA1c (mmol/mol) | 55.0±10.1 | 56.5±11.3 | 55.4±8.6 | 0.865 | 0.841 | 0.755 |  |
| GMI (%) | 7.1±0.7 | 7.3±0.7 | 6.9±0.3 | 0.384 | 0.279 | 0.293 |  |
| GMI (mmol/mol) | 54.6±7.2 | 56.0±7.1 | 52.5±3.8 | 0.407 | 0.307 | 0.309 |  |
| TIR (%) | 65.6±17.8 | 62.8±14.5 | 68.6±14.5 | 0.511 | 0.292 | 0.140 |  |
| TAR_>180mg/dl_ (%) | 31.2±17.6 | 34.7±16.1 | 29.5±14.5 | 0.745 | 0.515 | 0.204 |  |
| TBR_<70mg/dl_ (%) | 3.2±3.5 | 2.6±4.3 | 1.9±1.4 | 0.184 | 0.411 | 0.512 |  |
| Therapy (M/OL/CL) | 6/8/19 | 6/9/19 | 8/14/11 |  | 0.285 |  |  |
| Lipid lowering drugs (%) | 21.2 | 26.5 | 33.3 |  | 0.496 |  |  |
| Antihypertensive drugs (%) | 15.2 | 26.5 | 18.2 |  | 0.412 |  |  |
| **WOMEN** | | | | | | | |
|  | **Low tertile**  **(<291.2** **µmol/L) (n=33)** | **Medium tertile**  **(291.2-358.5** **µmol/L) (n=33)** | **High tertile**  **(> 358.5 µmol/L)**  **(n=32)** | **p for trend** | **p-value**  **ANOVA** | **p-value**  **adjusted for age and BMI** | |
| Age (Years) | 41.3±14.4 | 33.9±11.9 | 37.5±12.0 | 0.245 | 0.075 |  |  |
| BMI (Kg/m^2^) | 25.0±4.9 | 24.4±3.0 | 26.6±35.8 | 0.187 | 0.189 |  |  |
| HbA1c (%) | 7.9±1.1 | 7.5±0.9 | 7.7±0.8 | 0.438 | 0.291 | 0.508 |  |
| HbA1c (mmol/mol) | 63.0±11.2 | 58.2±9.6 | 60.3±8.1 | 0.343 | 0.200 | 0.376 |  |
| GMI (%) | 7.2±0.5 | 7.4±0.6 | 7.3±0.7 | 0.609 | 0.628 | 0.782 |  |
| GMI (mmol/mol) | 55.5±5.2 | 57.7±7.2 | 56.8±7.7 | 0.606 | 0.615 | 0.782 |  |
| TIR (%) | 63.8±15.5 | 58.8±16.1 | 61.9±16.3 | 0.664 | 0.527 | 0.776 |  |
| TAR_>180mg/dl_ (%) | 33.6±15.8 | 39.0±17.4 | 36.5±16.4 | 0.546 | 0.512 | 0.747 |  |
| TBR_<70mg/dl_ (%) | 2.5±2.1 | 2.2±4.1 | 2.1±2.7 | 0.608 | 0.142 | 0.832 |  |
| Therapy (M/OL/CL) | 5/11/17 | 7/14/12 | 5/11/16 |  | 0.819 |  |  |
| Lipid lowering drugs (%) | 30.3 | 12.1 | 28.1 |  | 0.186 |  |  |
| Antihypertensive drugs (%) | 12.1 | 6.1 | 12.5 |  | 0.653 |  |  |
| Data are expressed as mean ± SD. ^a^p<0.05 vs. Low tertile, Bonferroni post-hoc analysis. BMI, Body Mass Index; CL, Closed loop; GMI, glucose management indicator; HbA1c, glycated haemoglobin; M, multiple daily injections; OL, Open loop; TAR, Time Above Range; TBR, Time Below Range; TIR, Time In Range. | | | | | | | |
