# Supplementary material for: Sex-specific associations of serum short-chain fatty acids with glycaemic control: an Italian cross-sectional study in adults with type 1 diabetes
Source: BMJ Open. 2025 Mar 24;15(3):e096994. doi: 10.1136/bmjopen-2024-096994 (PMC11934402; doi:10.1136/bmjopen-2024-096994)
Supplement: online supplemental file 2 [file bmjopen-15-3-s002.docx]

| **Supplementary table 2.** Anthropometric parameters, therapy, and blood glucose control according to butyric acid tertiles stratifying the cohort by sex. | | | | | | |  |
| --- | --- | --- | --- | --- | --- | --- | --- |
| **MEN** | | | | | | |  |
|  | **Low tertile**  **(<17.5 μmol/L)**  **(n=33)** | **Medium tertile**  **(17.5-23.2** **μmol/L) (n=35)** | **High tertile**  **(> 23.2** **μmol/L)**  **(n=32)** | **p for trend** | **p-value**  **ANOVA** | **p-value adjusted for age and BMI** | |
| Age (Years) | 41.1±14.6 | 39.4±13.7 | 39.3±15.1 | 0.545 | 0.808 |  | |
| BMI (Kg/m^2^) | 26.7±4.8 | 25.7±3.2 | 25.7±3.1 | 0.334 | 0.545 |  | |
| HbA1c (%) | 7.3±1.0 | 7.0±0.8 | 7.5±0.9 | 0.346 | 0.084 | 0.126 | |
| HbA1c(mmol/mol) | 55.9±10.8 | 52.8±8.6 | 58.5±9.9 | 0.328 | 0.082 | 0.124 | |
| GMI (%) | 7.1±0.7 | 7.2±0.5 | 7.2±0.5 | 0.511 | 0.711 | 0.788 | |
| GMI (mmol/mol) | 53.8±7.3 | 54.8±5.8 | 54.6±6.4 | 0.572 | 0.646 | 0.757 | |
| TIR_70-180mg/dl_ (%) | 63.2±80.1 | 67.3±14.8 | 65.7±14.1 | 0.455 | 0.739 | 0.692 | |
| TAR_>180mg/dl_ (%) | 34.3±19.2 | 30.0±14.7 | 31.6±14.3 | 0.579 | 0.640 | 0.639 | |
| TBR_<70mg/dl_ (%) | 2.5±4.4 | 2.6±3.4 | 2.6±1.8 | 0.943 | 0.996 | 0.939 | |
| Therapy (M/OL/CL) | 7/8/18 | 8/12/18 | 8/11/13 |  | 0.285 |  | |
| Lipid lowering drugs (%) | 27.0 | 20.0 | 34.4 |  | 0.535 |  | |
| Antihypertensive drugs (%) | 27.3 | 17.1 | 15.6 |  | 0.485 |  | |
| **WOMEN** | | | | | | |  |
|  | **Low tertile (<18.1 μmol/L) (n=33)** | **Medium tertile**  **(18.1-24.8** **μmol/L) (n=33)** | **High tertile**  **(>24.8** **μmol/L) (n=32)** | **p for trend** | **p-value**  **ANOVA** | **p-value adjusted for age and BMI** | |
| Age (Years) | 36.5±11.1 | 38.9±13.1 | 37.7±15.2 | 0.720 | 0.770 |  | |
| BMI (Kg/m^2^) | 24.5±4.4 | 26.1±5.1 | 25.6±4.8 | 0.353 | 0.400 |  | |
| HbA1c (%) | 7.4±0.7 | 7.8±0.9 | 7.8±0.9 | 0.101 | 0.208 | 0.343 | |
| HbA1c (mmol/mol) | 58.1±7.4 | 61.4±10.5 | 62.0±10.8 | 0.128 | 0.258 | 0.418 | |
| GMI (%) | 7.4±0.6 | 7.2±0.5 | 7.3.±0.7 | 0.436 | 0.573 | 0.416 | |
| GMI (mmol/mol) | 57.8±6.4 | 55.6±5.5 | 56.5±8.1 | 0.448 | 0.554 | 0.413 | |
| TIR_70-180 mg/dl_ (%) | 59.5±14.9 | 66.0±13.2 | 60.2±18.9 | 0.864 | 0.287 | 0.166 | |
| TAR_>180mg/dl_ (%) | 38.2±15.2 | 31.7±14.3 | 37.9±19.4 | 0.981 | 0.300 | 0.179 | |
| TBR_<70mg/dl_ (%) | 2.5±2.8 | 2.6±4.3 | 1.9±1.3 | 0.482 | 0.645 | 0.800 | |
| Therapy (M/OL/CL) | 8/11/14 | 3/12/18 | 5/13/14 |  | 0.657 |  | |
| Lipid lowering drugs (%) | 12.1 | 33.3 | 25 |  | 0.139 |  | |
| Antihypertensive drugs (%) | 6.1 | 12.1 | 12.5 |  | 0.653 |  | |
| Data are expressed as mean ± SD. ^a^p<0.05 vs. Low tertile, Bonferroni post-hoc analysis. BMI, Body Mass Index; CL, Closed loop; GMI, glucose management indicator; HbA1c, glycated haemoglobin; M, multiple daily injections; OL, Open loop; TAR, Time Above Range; TBR, Time Below Range; TIR, Time In Range. . | | | | | | |  |
