# Supplementary material for: Sex-specific associations of serum short-chain fatty acids with glycaemic control: an Italian cross-sectional study in adults with type 1 diabetes
Source: BMJ Open. 2025 Mar 24;15(3):e096994. doi: 10.1136/bmjopen-2024-096994 (PMC11934402; doi:10.1136/bmjopen-2024-096994)
Supplement: online supplemental file 3 [file bmjopen-15-3-s003.docx]

| **Supplementary table 3.** Daily dietary composition (7-day food diary) according to Propionic acid tertiles stratifying the cohort by sex | | | | | | |
| --- | --- | --- | --- | --- | --- | --- |
| **MEN** | | | | | | |
|  | **Low tertile**  **(<23.1 µmol/L)**  **(n=23)** | **Medium tertile**  **(23.1-30.8 µmol/L) (n=24)** | **High tertile**  **(>30.8** **µmol/L) (n=22)** | **p for trend** | **p-value**  **ANOVA** | **p-value adjusted for age and BMI** |
| Energy (kcal) | 1534±378 | 1597±295 | 1625±524 | 0.455 | 0.744 | 0.567 |
| Protein (%) | 20.6±2.6 | 20.0±3.2 | 19.6±4.8 | 0.347 | 0.639 | 0.562 |
| - Vegetables (%) | 6.8±1.1 | 6.8±1.8 | 6.9±1.4 | 0.833 | 0.975 | 0.988 |
| - Animals (%) | 12.2±2.5 | 11.9±3.0 | 11.1±5.1 | 0.292 | 0.558 | 0.549 |
| Total Fat (%) | 34.9±5.1 | 36.4±7.9 | 35.6±6.8 | 0.732 | 0.735 | 0.722 |
| - SFA (%) | 11.4±2.9 | 11.7±4.7 | 10.2±2.3 | 0.280 | 0.344 | 0.371 |
| - MUFA (%) | 15.8±3.3 | 17.2±4.9 | 16.6±4.9 | 0.534 | 0.561 | 0.546 |
| - PUFA (%) | 4.2±1.2 | 4.5±1.1 | 4.3±0.9 | 0.726 | 0.634 | 0.606 |
| Carbohydrates (%) | 44.8±5.1 | 43.8±6.9 | 45±7.2 | 0.935 | 0.791 | 0.864 |
| Simple sugars (%) | 12.3±4.4 | 12.6±5.8 | 10.7±4.8 | 0.322 | 0.420 | 0.433 |
| Fiber (g/1000 kcal) | 10.5±2.9 | 11.3±4.2 | 9.6±2.3 | 0.381 | 0.240 | 0.205 |
| **WOMEN** |  | | | | | |
|  | **Low tertile**  **(<25.1** **µmol/L)**  **(n=26)** | **Medium tertile**  **(25.1-32.1** **µmol/L) (n=22)** | **High tertile**  **(>32.1** **µmol/L) (n=20)** | **p for trend** | **p-value**  **ANOVA** | **p-value adjusted for age and BMI** |
| Energy (kcal) | 1362±313 | 1361±294 | 1251±281 | 0.486 | 0.752 | 0.437 |
| Protein (%) | 18.8±3.0 | 19.1±2.5 | 20.4±3.9 | 0.096 | 0.195 | 0.152 |
| - Vegetables (%) | 6.4±1.7 | 7.4±2.3 | 7.1±1.6 | 0.206 | 0.201 | 0.300 |
| - Animals (%) | 10.9±3.7 | 10.4±3.2 | 11.4±4.2 | 0.699 | 0.700 | 0.546 |
| Total Fat (%) | 39.1±5.0 | 34.9±5.6^a^ | 37.8±4.9 | 0.281 | **0.022** | **0.019** |
| - SFA (%) | 11.9±2.4 | 10.4±2.5 | 11.0±2.9 | 0.168 | 0.104 | 0.086 |
| MUFA (%) | 17.8±3.2 | 16.2±3.9 | 18.4±3.4 | 0.701 | 0.123 | 0.060 |
| - PUFA (%) | 4.9±1.4 | 4.4±1.1 | 4.9±0.8 | 0.824 | 0.306 | 0.434 |
| Carbohydrates (%) | 42.5±5.9 | 46.2±6.3 | 41.9±5.5^b^ | 0.722 | **0.040** | **0.027** |
| Simple Sugars (%) | 12.6±4.1 | 13.0±4.6 | 12.4±4.8 | 0.911 | 0.880 | 0.911 |
| Fiber (g/1000 kcal) | 11.4±3.6 | 12.3±4.2 | 12.7±4.4 | 0.286 | 0.559 | 0.760 |
| Data are expressed as mean ± SD. Bonferroni post-hoc analysis, ^a^p<0.05 vs Low tertile ^b^p<0.05 vs Medium tertile. MUFA, monounsaturated fatty acids; PUFA, polyunsaturated fatty acids, SFA, Saturated fatty acids. | | | | | | |
