# Supplementary material for: Sex-specific associations of serum short-chain fatty acids with glycaemic control: an Italian cross-sectional study in adults with type 1 diabetes
Source: BMJ Open. 2025 Mar 24;15(3):e096994. doi: 10.1136/bmjopen-2024-096994 (PMC11934402; doi:10.1136/bmjopen-2024-096994)
Supplement: online supplemental file 4 [file bmjopen-15-3-s004.docx]

| **Supplementary table 4.** Daily dietary composition (7-day-food diary) according to butyric acid tertiles stratifying the cohort by sex | | | | | | | | | | |
| --- | --- | --- | --- | --- | --- | --- | --- | --- | --- | --- |
| **MEN** | | | | | | | | | | |
|  | **Low tertile**  **(<17.5 µmol/L) (n=21)** | | | | **Medium tertile**  **(17.5-23.2 µmol/L)**  **(n=26)** | **High tertile**  **(>23.2** **µmol/L) (n=22)** | **p for trend** | **p-value**  **ANOVA** | **p-value adjusted for age and BMI** |  |
| Energy (kcal) | | | 1583±366 | | 1543±387 | 1634±448 | 0.672 | 0.736 | 0.785 |  |
| Protein (%) | | | 21±3.7 | | 29.4±3.6 | 20±3.3 | 0.347 | 0.315 | 0.349 |  |
| - Vegetables (%) | | | 12.3±3.4 | | 11.3±3.9 | 11.7±3.6 | 0.451 | 0.625 | 0.561 |  |
| - Animals (%) | | | 7.1±1.3 | | 6.7±1.4 | 6.8±1.6 | 0.609 | 0.654 | 0.711 |  |
| Total Fat (%) | | | 33±6.1 | | 38±6.6 ^a^ | 34.9±6.6 | 0.371 | **0.037** | **0.037** |  |
| - SFA (%) | | | 10.7±4.8 | | 11.8±2.8 | 10.7±2.8 | 0.992 | 0.446 | 0.489 |  |
| - MUFA (%) | | | 15.6±4.4 | | 17.6±4.4 | 16±4.2 | 0.816 | 0.258 | 0.158 |  |
| - PUFA (%) | | | 4.4±1.1 | | 4.4±1.2 | 4.2±0.9 | 0.529 | 0.814 | 0.847 |  |
| Carbohydrates (%) | | | 46.3±5.6 | | 42.7±6.1 | 45.4±7.1 | 0.689 | 0.128 | 0.117 |  |
| Simple sugars (%) | | | 13.5±6.4 | | 10.3±3.6 | 12.8±4.8 | 0.675 | 0.075 | 0.217 |  |
| Fiber (g/1000 kcal) | | | 11.5±3.4 | | 10.1±2.6 | 10.4±4.0 | 0.293 | 0.330 | 0.655 |  |
| **WOMEN** | | | | | | | | | | |
|  | | **Low tertile**  **(<18.1** **µmol/L)**  **(n=24)** | | | **Medium tertile**  **(18.1-24.8 µmol/L)**  **(n=24)** | **High tertile**  **(> 24.8 µmol/L) (n=20)** | **p for trend** | **p-value**  **ANOVA** | **p-value adjusted for age and BMI** |  |
| Energy (kcal) | | | | 1252±274 | 1335±298 | 1264±329 | 0.837 | 0.593 | 0.389 |  |
| Protein (%) | | | | 19.7±3.5 | 18.7±2.3 | 19.8±3.3 | 0.930 | 0.392 | 0.392 |  |
| - Vegetables (%) | | 7.0±0.8 | | | 7.1±1.7 | 6.5±1.7 | 0.431 | 0.633 | 0.578 |  |
| - Animals (%) | | 11.0±4.4 | | | 10.3±2.5 | 11.7±3.9 | 0.557 | 0.460 | 0.508 |  |
| Total Fat (%) | | 36.5±5.8 | | | 38.3±4.7 | 37.2±5.8 | 0.632 | 0.503 | 0.450 |  |
| - SFA (%) | | 11.2±2.5 | | | 11.2±2.2 | 11.2±3.4 | 0.976 | 0.998 | 0.880 |  |
| - MUFA (%) | | 16.3±3.6 | | | 17.5±3.0 | 17.4±3.6 | 0.230 | 0.150 | 0.261 |  |
| - PUFA (%) | | 4.6±1.3 | | | 5.03±1.3 | 4.6±0.80 | 0.913 | 0.300 | 0.275 |  |
| Carbohydrates (%) | | | 44.2±7.0 | | 43.3±4.4 | 43.1±7.0 | 0.548 | 0.811 | 0.904 |  |
| Simple sugars (%) | | | 12.7±4.1 | | 12.1±3.9 | 13.1±5.4 | 0.823 | 0.777 | 0.501 |  |
| Fiber (g/1000 kcal) | | | 13.2±5.1 | | 12.0±3.4 | 11.2±4.5 | 0.130 | 0.306 | 0.172 |  |
| Data are expressed as mean ± SD. ^a^p<0.05 vs. Low tertile. Bonferroni post-hoc analysis. MUFA, monounsaturated fatty acids; PUFA, polyunsaturated fatty acids, SFA, saturated fatty acids. | | | | | | | | | | |
