# Supplementary material for: Sex-specific associations of serum short-chain fatty acids with glycaemic control: an Italian cross-sectional study in adults with type 1 diabetes
Source: BMJ Open. 2025 Mar 24;15(3):e096994. doi: 10.1136/bmjopen-2024-096994 (PMC11934402; doi:10.1136/bmjopen-2024-096994)
Supplement: Supplementary file 5 [file bmjopen-2024-096994.reviewer_comments.pdf]

PEER REVIEW HISTORY

BMJ Open publishes all reviews undertaken for accepted manuscripts. Reviewers are asked to complete a checklist review form and are provided with free text boxes to elaborate on their assessment. These free text comments are reproduced below.

ARTICLE DETAILS

Title (Provisional)

Sex-Specific Associations of Serum Short-Chain Fatty Acids with Glycaemic Control: An Italian Cross-sectional Study in Adults with Type 1 Diabetes

Authors

Abuqwider, Jumana; Salamone, Dominic; Scidà, Giuseppe; Corrado, Alessandra; Costabile, Giuseppina; Luongo, Delia; Annuzzi, Giovanni; Rivellese, Angela; Bozzetto, Lutgarda

---

VERSION 1 - REVIEW

**Reviewer**	**1**
**Name**	**Mendivil, Carlos O**
**Affiliation**	**Universidad de los Andes, Medicine**
**Date**	**04-Jan-2025**
**COI**	**None**

This is an interesting paper about the association of plasma concentrations of short-chain fatty acids, presumably originated by the gut microbiota, and various measures of glycemic control among patients with type 1 diabetes. The design is simple but clear and appropriately addresses the research questions, I have only minor comments:

Abstract:

1.Always define abbreviations on first use

2. At least briefly mention why it would be of interest to look at SCFA in patients with T1DM, beyond the fact that it hasn´t been done before.

3. Results section: Show the magnitude of the differences, not just the p-value.

4. Chack the writing of the last sentence of the conclusion

Intro

5. Please italicize in vivo and in vitro

Methods

6. Briefly mention which CGM devices were used by study participants

7. Please use CSII instead of "insulin pumps"

8. Please report on the procedures to estimate nutrient intakes from the food diary data

9. When was the blood sample for measurement of SCFA drwn, relative to the period of calculation of CGM measures (TIR, TAR, etc)?

10. Please provide more detail on the analytical procedure for SCFA determination including extraction, internal and/or external standards, and quality control measures

Results

11. In the text, briefly mention the magnitude of differences, not just the p-values

Discussion

12. Page 8, lines 4-5, use either....or

13. Please mention why is a 7% difference in serum propionate clinically meaningful. Also, this figure seems different from what apears in Table 2

14. Please don´t talk of a beneficial effect of propionate on glycemic control, this is not supported by the study design

15. The argument presented in page 8, lines 26-27 does not seem to be supported by the data in Table 2

Table 2

16. No need to show p-values for the partially adjusted models, just for the fully adjusted model

17. No need to report in the footnotes the results from Bonferroni-adjusted post-hoc tests, as these are supposed to be reported only when the global ANOVA is significant

Figure 1

18. Please specify what the error bars and box sizes represent

---

**Reviewer**	**2**
**Name**	**Snaith, Jennifer**
**Affiliation**	**Garvan Institute of Medical Research**
**Date**	**19-Jan-2025**
**COI**	**None**

This was a cross-sectional study examining relationships between serum SCFA and glycemia in adults with T1D. The main conclusion was that in women, propionate was associated with time in range. This manuscript was written clearly and adds novelty, as there is paucity of knowledge in T1D with regards to relationships between SCFA and metabolism. This study raises new hypotheses about the role of propionate on metabolism in T1D.

Minor comments

- Introduction paragraph 2 – the relevance of SCFA to metabolism is detailed here very nicely, though suggest the authors expand this background specify from which populations this existing knowledge is based (eg. lean non-diabetic, type 2 diabetes, type 1 diabetes)

- Associations were determined by dividing participants into tertiles for SCFA levels. Did the authors consider analysing the data on a continuum eg. using pearsons or spearmans?

- Table 1 – typo 'Hb1Ac'

- Discussion line 35-36: could it also be said that we cannot exclude the possibility that increases in propionate levels could lead to improvements in glycaemia

- Could the authors discuss possible explanations for the demonstration of associations between CGM indices and propionate, without an association with HbA1c? How acutely do SCFA levels change, and would serum SCFA be more reflective of recent glycaemia captured by CGM?

Re: review checklist - was unable to tick yes for STROBE checklist complete

---

VERSION 1 - AUTHOR RESPONSE

Reviewer: 1

Dr. Carlos O Mendivil, Universidad de los Andes, Fundacion Santa Fe de Bogota

Comments to the Author:

This is an interesting paper about the association of plasma concentrations of short-chain fatty acids, presumably originated by the gut microbiota, and various measures of glycemic control among patients with type 1 diabetes. The design is simple but clear and appropriately addresses the research questions, I have only minor comments:

Abstract:

1. Always define abbreviations on first use.

Re. Done. Thank you.

2. At least briefly mention why it would be of interest to look at SCFA in patients with T1DM, beyond the fact that it hasn´t been done before.

Re. A sentence has been added to make it clearer (page 2, lines 3-4)

3. Results section: Show the magnitude of the differences, not just the p-value.

Re. The absolute data have been added (page 2, lines 15-17)

4. Chack the writing of the last sentence of the conclusion

Re. The last sentence has been rephrased (page 2, lines 17-19)

Intro

5. Please italicize in vivo and in vitro

Re. Done

Methods

6. Briefly mention which CGM devices were used by study participants

Re. The types of CGM used by the participants has been now added (page 5, lines 12-16).

7. Please use CSII instead of "insulin pumps"

Re. Changed as suggested (page 4, line 36)

8. Please report on the procedures to estimate nutrient intakes from the food diary data

Re: As suggested, this info has been added in the new version (page 4, lines 40-42; page 5, lines 1-2).

9. When was the blood sample for measurement of SCFA drown, relative to the period of calculation of CGM measures (TIR, TAR, etc)?

Re. Blood samples were taken on the day of the annual visit for the screening of complications (page 5, line 7) and data from CGM devices refer to the two weeks preceding the visit (page 5, lines 12-16)

10. Please provide more detail on the analytical procedure for SCFA determination including extraction, internal and/or external standards, and quality control measures

Re. We added more details on the analytic procedure in the Materials and Methods section (page 5, lines 23-30).

Results

11. In the text, briefly mention the magnitude of differences, not just the p-values

Re. The magnitude of differences has been added in the Results section (page 6, lines 18-20)

Discussion

12. Page 8, lines 4-5, use either....or

Re. Changed in the new version.

13. Please mention why is a 7% difference in serum propionate clinically meaningful. Also, this figure seems different from what apears in Table 2

Re. It has been clarified that the 9% difference refers to TIR and not propionate (page 7,lines 4-8)

14. Please don´t talk of a beneficial effect of propionate on glycemic control, this is not supported by the study design

Re. The sentence has been removed in the new version..

15. The argument presented in page 8, lines 26-27 does not seem to be supported by the data in Table 2

Re. The argument presented in page 8, lines 26-27 is one of the possible explanations of the sex differences and refers to data presented in table 1. It has been rephrased to make it clearer (page 7 lines 14-23)

Table 2

16. No need to show p-values for the partially adjusted models, just for the fully adjusted model

Re. As suggested, we removed the columns presenting p-values for the partially adjusted models (tables 2 and 4 and supplementary tables 1-4)

17. No need to report in the footnotes the results from Bonferroni-adjusted post-hoc tests, as these are supposed to be reported only when the global ANOVA is significant

Re. Now, the results of the Bonferroni post-hoc test are reported in the footnotes only when ANOVA is significant.

Figure 1

18. Please specify what the error bars and box sizes represent

Re. The custom boxplots in Figures 1 and 2 display the mean as the central line, with the lower and upper bounds of the box set one standard deviation below and above the mean. The whiskers extend from the minimum to the maximum values, representing the full range of the dataset. This is now defined in the figure legends.

Reviewer: 2

Dr. Jennifer Snaith, Garvan Institute of Medical Research

Comments to the Author:

This was a cross-sectional study examining relationships between serum SCFA and glycemia in adults with T1D. The main conclusion was that in women, propionate was associated with

time in range. This manuscript was written clearly and adds novelty, as there is paucity of knowledge in T1D with regards to relationships between SCFA and metabolism. This study raises new hypotheses about the role of propionate on metabolism in T1D.

Minor comments

- Introduction paragraph 2 – the relevance of SCFA to metabolism is detailed here very nicely, though suggest the authors expand this background specify from which populations this existing knowledge is based (eg. lean non-diabetic, type 2 diabetes, type 1 diabetes)

Re. We added this information in the Introduction section (page 4, lines 4-6).

- Associations were determined by dividing participants into tertiles for SCFA levels. Did the authors consider analysing the data on a continuum eg. using pearsons or spearmans?

Re. We have done also correlation analysis, and the results go in the same direction. We preferred using tertiles because it is clearer, can help identify patterns or trends, and makes easier to spot differences between groups.

- Table 1 – typo 'Hb1Ac'

Re. Thank you. The typo has been corrected.

- Discussion line 35-36: could it also be said that we cannot exclude the possibility that increases in propionate levels could lead to improvements in glycaemia

Re. We agree with the reviewer that this remains the more likely explanation for the association although the causal relationship cannot be established by our results due to the cross-sectional design of the study (page 7, lines 21-23).

- Could the authors discuss possible explanations for the demonstration of associations between CGM indices and propionate, without an association with HbA1c? How acutely do SCFA levels change, and would serum SCFA be more reflective of recent glycemia captured by CGM?

Re. As suggested by the reviewer, the associations with CGM indices and not with HbA1c values could be explained by the different durations of the two measurements, CGM indices being able to capture short-term fluctuations missed by HbA1c measurements (page 7, line 6-8). It is also possible a role for rapid changes of SCFA levels, but, as far as we know, no data are available on this issue.

Re: review checklist - was unable to tick yes for STROBE checklist complete (?)We added strobe check list

VERSION 2 - REVIEW

**Reviewer**	**1**
**Name**	**Mendivil, Carlos O**

Affiliation **Universidad de los Andes, Medicine**

Date **03-Mar-2025**

COI

All of my comments have been successfully addressed
